# Supplementary material for: Modulation of Pro-Inflammatory IL-6 Trans-Signaling Axis by Splice Switching Oligonucleotides as a Therapeutic Modality in Inflammation
Source: Cells. 2023 Sep 15;12(18):2285. doi: 10.3390/cells12182285 (PMC10526877; doi:10.3390/cells12182285)
Supplement: Supplementary file 1 [file cells-12-02285-s001.zip › cells-2561440-supplementary.pdf]

Supplementary information

A

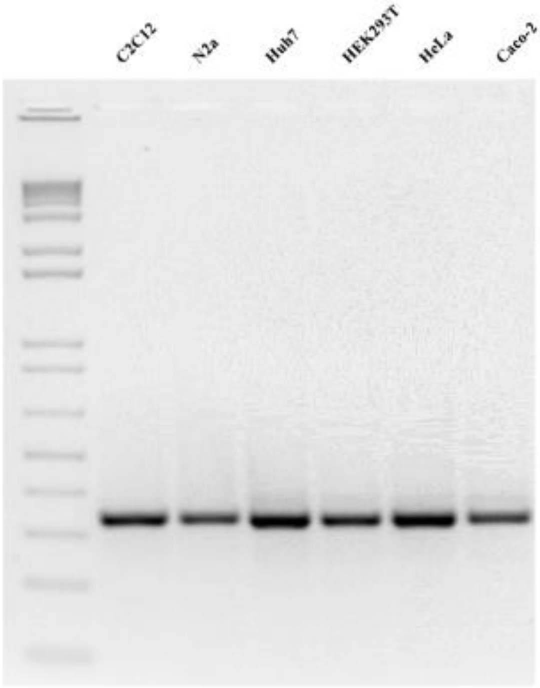

Figure S1: Expression of Gp130 RNA was checked in mouse C2C12, mouse N2A, human Huh7, human HEK293T, human HeLa, and human Caco-2. 2% Agarose gel image showing total RNA RT-PCR using primers targeting exon 8 and exon 10 of Gp130, where the 198 bp band corresponds to the Gp130 isoform with no exon skipping in this region.

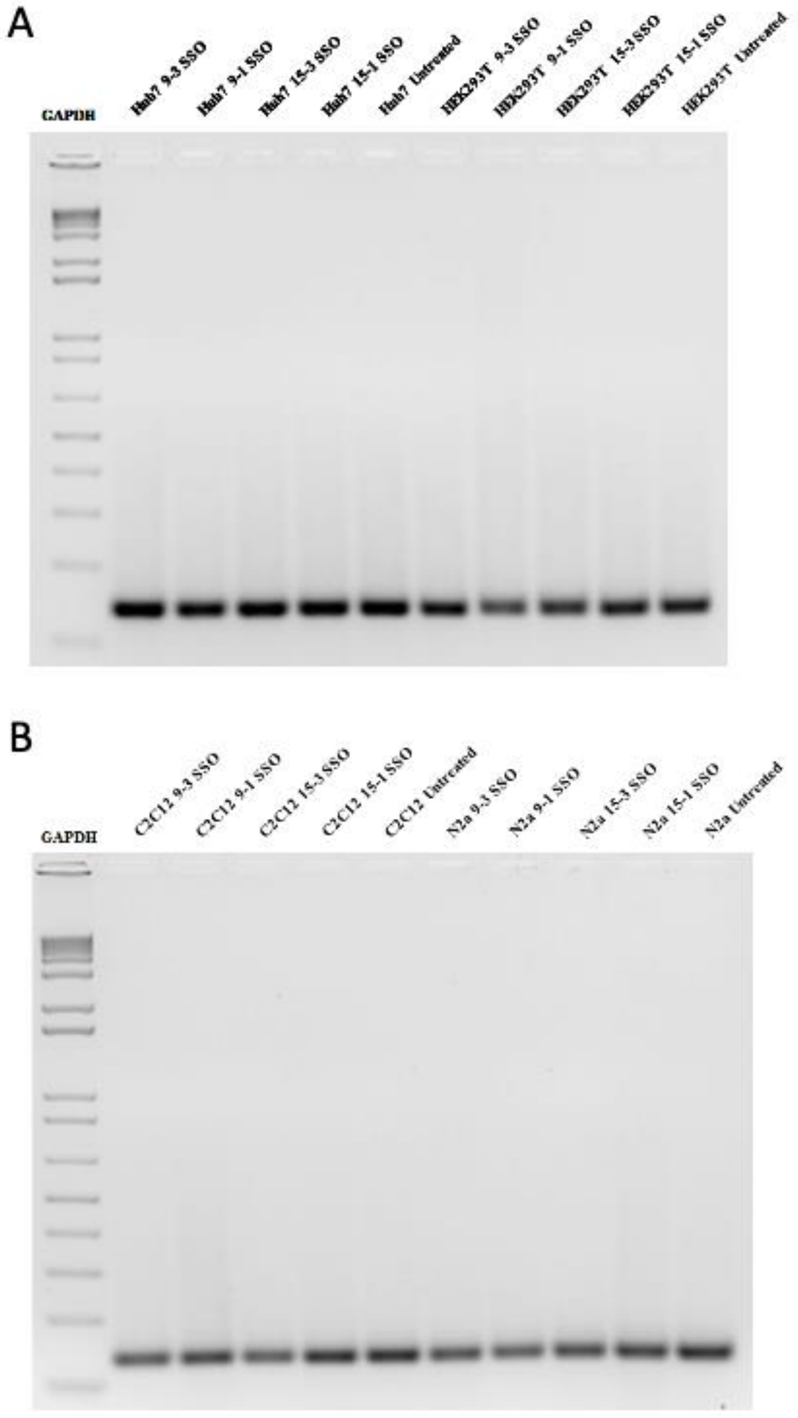

Figure S2: RT-PCR showing amplification of GAPDH mRNA in RNA samples from cells treated with different SSOs.

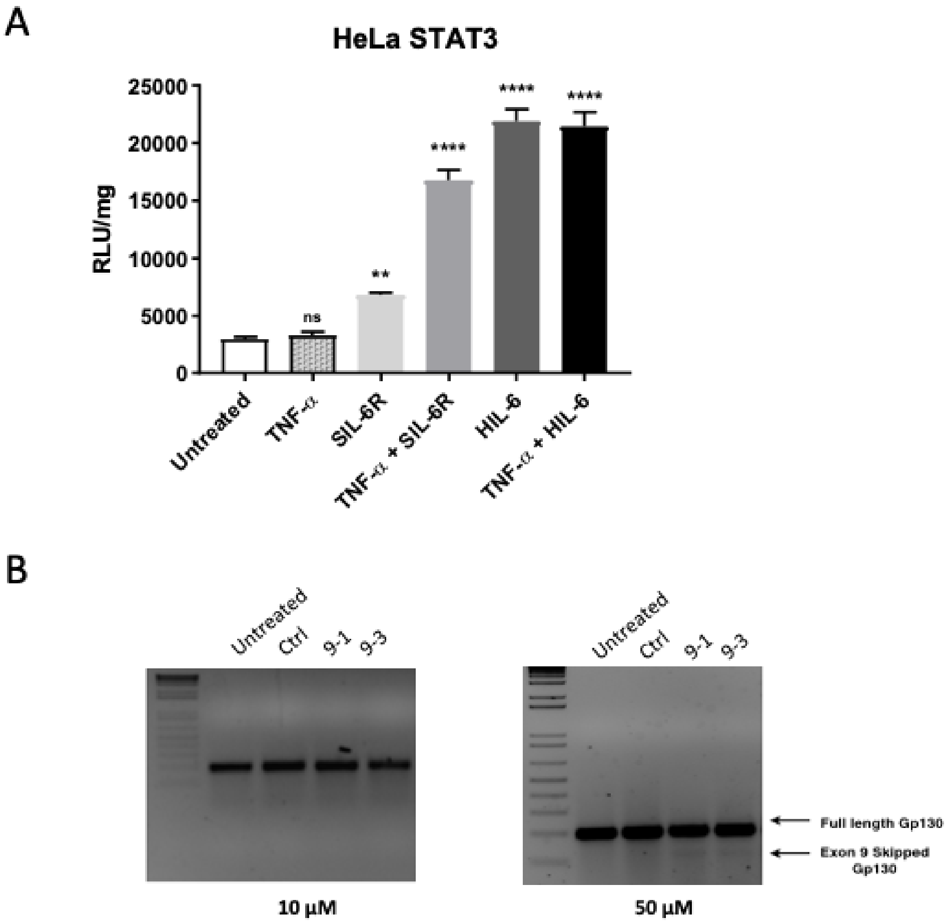

Figure S3: A) Relative Light Unit RLU/mg protein in HeLa STAT3 Luciferase reporter cell line, induced by 5ng/ml TNFα, 20 ng/ml SIL-6R and/or 10ng/ml HIL-6. Statistical analysis was done with One-Way ANOVA and Dunnett's multiple comparisons test. Data represent mean + SD of three replicates. **** ($P < 0.0001$), ** ($P < 0.001$), ns ($P > 0.05$) – not significant, versus untreated. B) Gymnotic delivery of PMO-modified Exon 9 SSOs showed modest exon skipping in mouse N2A cells. RT-PCR showing exon 9 skipping efficiency of Gp130 mRNA in RNA samples.
